# Genome of lethal *Lepiota venenata* and insights into the evolution of toxin-biosynthetic genes

**DOI:** 10.1186/s12864-019-5575-7

**Published:** 2019-03-08

**Authors:** Yunjiao Lüli, Qing Cai, Zuo H. Chen, Hu Sun, Xue-Tai Zhu, Xuan Li, Zhu L. Yang, Hong Luo

**Affiliations:** 1 CAS Key Laboratory for Plant Diversity and Biogeography of East Asia, Kunming Institute of Botany, Chinese Academy of Sciences, Kunming, 650201 Yunnan China; 2 College of Life Science, Hunan Normal University, Changsha, 410081 China; 3 College of Life Sciences, Northwest Normal University, Lanzhou, 730030 China; 4 Department of Environmental Science and Engineering, Kunming University of Science and Technology, Kunming, 650091 Yunnan China; 5 University of Chinese Academy of Sciences, Beijing, 100049 China

**Keywords:** Genome, *Lepiota*, Amanitin, Phylogeny, Horizontal gene transfer

## Abstract

**Background:**

Genomes of lethal *Amanita* and *Galerina* mushrooms have gradually become available in the past ten years; in contrast the other known amanitin-producing genus, *Lepiota*, is still vacant in this aspect. A fatal mushroom poisoning case in China has led to acquisition of fresh *L. venenata* fruiting bodies, based on which a draft genome was obtained through PacBio and Illumina sequencing platforms. Toxin-biosynthetic MSDIN family and Porlyl oligopeptidase B (*POPB*) genes were mined from the genome and used for phylogenetic and statistical studies to gain insights into the evolution of the biosynthetic pathway.

**Results:**

The analysis of the genome data illustrated that only one MSDIN, named *LvAMA1*, exits in the genome, along with a *POPB* gene. No *POPA* homolog was identified by direct homology searching, however, one additional *POP* gene, named *LvPOPC*, was cloned and the gene structure determined. Similar to *ApAMA1* in *A. phalloides* and *GmAMA1* in *G. marginata*, *LvAMA1* directly encodes α-amanitin. The two toxin genes were mapped to the draft genome, and the structures analyzed. Furthermore, phylogenetic and statistical analyses were conducted to study the evolution history of the *POPB* genes. Compared to our previous report, the phylogenetic trees unambiguously showed that a monophyletic *POPB* lineage clearly conflicted with the species phylogeny. In contrast, phylogeny of *POPA* genes resembled the species phylogeny. Topology and divergence tests showed that the *POPB* lineage was robust and these genes exhibited significantly shorter genetic distances than those of the house-keeping *rbp2*, a characteristic feature of genes with horizontal gene transfer (HGT) background. Consistently, same scenario applied to the only MSDIN, *LvAMA1*, in the genome.

**Conclusions:**

To the best of our knowledge, this is the first reported genome of *Lepiota*. The analyses of the toxin genes indicate that the cyclic peptides are synthesized through a ribosomal mechanism. The toxin genes, *LvAMA1* and *LvPOPB*, are not in the vicinity of each other. Phylogenetic and evolutionary studies suggest that HGT is the underlining cause for the occurrence of *POPB* and MSDIN in *Amanita*, *Galerina* and *Lepiota*, which are allocated in three distantly-related families.

**Electronic supplementary material:**

The online version of this article (10.1186/s12864-019-5575-7) contains supplementary material, which is available to authorized users.

## Background

α-Amanitin, perhaps the best known deadly mushroom toxin, is distributed in three disjunct genera, including *Amanita*, *Galerina* and *Lepiota* [1–6], which belong to distantly related families, namely Amanitaceae [7–11], Strophariaceae [12], and Agaricaceae [13], respectively. Whether *Conocybe* can produce similar cyclic peptide toxins is still in question [14, 15]. The bicyclic toxin is highly stable and resistant to high temperature, acids, alkalis and salts; general cooking methods do not destroy its toxicity [16]. Amanitins, including α-Amanitin, are synthesized through a ribosomal mechanism in *Amanita* and *Galerina* [17, 18]. However, the pathway of amanitin biosynthesis in *Lepiota* is unknown. With the advent of the genome era, a few genomes of lethal mushrooms containing α-amanitin have been published [17–20], but no genome of *Lepiota* species has been reported. In contrast to amanitin-producing agarics, genomes of other *Amanita* species including *A. muscaria* [21], *A. jacksonii* [22] and *A. thiersii* [23] have become available in recent years. These non-amanitin-producing *Amanita* species do not produce cyclic peptides and our extensive BLAST search in those genomes did not return any known toxin-biosynthetic genes, namely the MSDIN family and prolyl oligopeptidase B (*POPB*) genes.

In recent years, there have been many poisoning tragedies all over the world caused by accidental consumptions of poisonous mushrooms containing α-amanitin. It was reported in May 2018 that serious mushroom poisoning incidents occurred in Iran in a short period, which resulted in 18 deaths out of 1151 patients (http://www.tehrantimes.com/news/423947/Mushroom-poisoning-kills-18-in-Iran). *Amanita virosa*, a α-amanitin-containing species commonly known in Europe as the destroying angel, is likely responsible for this poisoning case. In lethal *Amanita* species, a mature individual may contain α-amanitin that exceeds the adult lethal dose [15, 24], and 144 deaths were caused by *Amanita* species in China during 1994–2016 [11]. In September 2017, two individuals died of *L. venenata* poisoning in Jingzhou City, Hubei Province of Central China [25]. After the incidence, we collected the fresh fruiting bodies of *L. venenata* from the locality, and these mushrooms were sent for genome sequencing with combined platform of PacBio Sequel and Illumina HiSeq X10. The draft genome was in turn used to study MSDIN and *POPB* genes that associate with amanitin biosynthesis. The biosynthesis of this toxin and related cyclic peptides begins with activation of MSDIN genes that encode a precursor peptide of 34–37 amino acids, with the first 5 highly conserved residues mostly being MSDIN [17]. The precursor peptides are cleaved and macrocyclized into 7–10 amino acid cyclic peptides by a specialized prolyl oligopeptidase, *POPB* [19, 26].

Our previous report found that the three disjunct genera, *Amanita*, *Galerina* and *Lepiota*, all possess a similar biosynthetic pathway for amanitin production, which is probably due to horizontal gene transfer (HGT) [14]. At the time, the phylogenetic approach did not fully rule out massive gene loss (over 2000 genes counted) as one of the possibilities. In this report, MSDIN and *POP* (*POPA* and *POPB*) genes were investigated based on a draft genome of the lethal *L. venenata*. The genes were mined and the structures determined. Using combined approaches, we found a new homolog of *POP* genes. With this new *POP* gene, we once again constructed phylogenetic trees of *POP* genes for more insights into the evolutionary history. Subsequently, the topology was examined, and the *POPB* branch tested for reliability. In this report, we mainly searched for conflict between *POPB* and species phylogenies, as this approach is considered as the most reliable way of assessing HGT. Other supporting methods, including genetic distance (applied to both *POPB* and MSDIN), topology test, and comparison of gene and species trees, were included to further support our conclusion.

## Results

### Overview of *L. venenata* genome and toxin genes

**Fig. 1:**
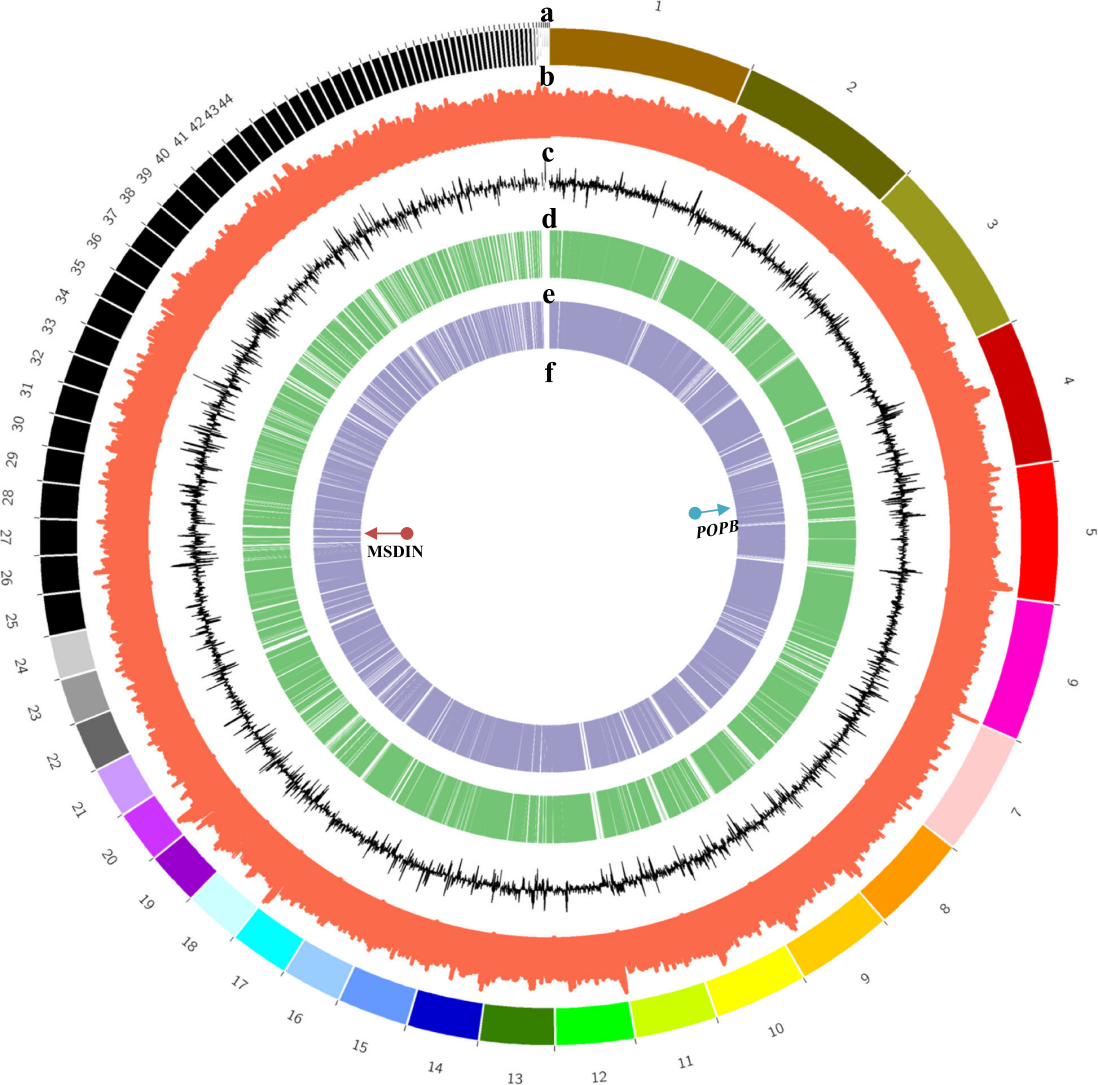
Circular map of genomic features of the *Lepiota venenata* genome. **a** Location of every contig. **b** GC content of the genome. **c** GC skew of the genome. **d** Location of predicted genes. **e** Exon positions of protein-coding genes. **f** Location of MSDIN and *POPB*

Genome sequencing of *L. venenata* was performed using single molecule real-time (SMRT) DNA sequencing. A draft genome sequence was generated on the PacBio Sequel platforms and Illumina HiSeq X10 (GenBank: RCFS00000000 Ver. RCFS01000000). The total amount of clean data was 4.23 Gb, and the size of assembled genome was 49.25 Mb. The main genomic features were shown in the table (Table 1). BUSCO prediction with Basidiomycota settings implied that 89.5% genes were found, and missing BUSCOs were at low level of 5.0%. Only one MSDIN gene was found after both tBLASTn and BLASTn searches using multiple queries from both *Amanita* and *Galerina*. This gene was located on contig 27, and in total we obtained 571 bp in length with 154 bp from ATG to TAA. The deduced amino acid sequence had 32 amino acids, and the 10th to the 17th amino acids, IWGIGCNP, coded for α-amanitin. The full amino acid sequence was MDANATRLP**IWGIGCNP**WTPESVNDTLTKDLS with the core peptide in bold and underlined. Due to the similarity to other *AMA1* genes, it was named *LvAMA1* (Additional file 1). The *POPB* gene (denoted as *LvPOPB* later) was found on contig 4 with 3118 bp in length (ATG to TGA) (Additional file 2). The genome was visualized as a circular diagram using Circos software (Fig. 1). The tracks (a to f) represented the contigs (a), GC content (b), GC skew (c), the location of predicted genes (d), the location of exons (e), and the distribution of the two toxin genes in the genome (f). As can be seen from the figure, the predicted distribution of genes and exons in the whole genome were relatively uniform. *LvPOPB* and *LvAMA1* were located on contigs 4 and 27 respectively, and were far apart (at least 525 Kb) with over 100 predicted genes in between. Clearly, these two toxin genes did not form a cluster.

**Table 1:** Genome features of *Lepiota venenata*

Genome features	Value
Size of assembled genome	49.25 Mb
Number of contigs	88
N50	1,272,972 bp
N90	344,036 bp
Maximum length	3,318,280 bp
GC content of assembled genome	49.02%
Total number of genes	13,686
Average gene length	1361.46 bp
Total number of CDS	57,930
Average CDS length	1104.54 bp
Number of Exons	57,930
Average exon length	259.94 bp

### Analysis of toxin(s) by LC-MS

**Fig. 2:**
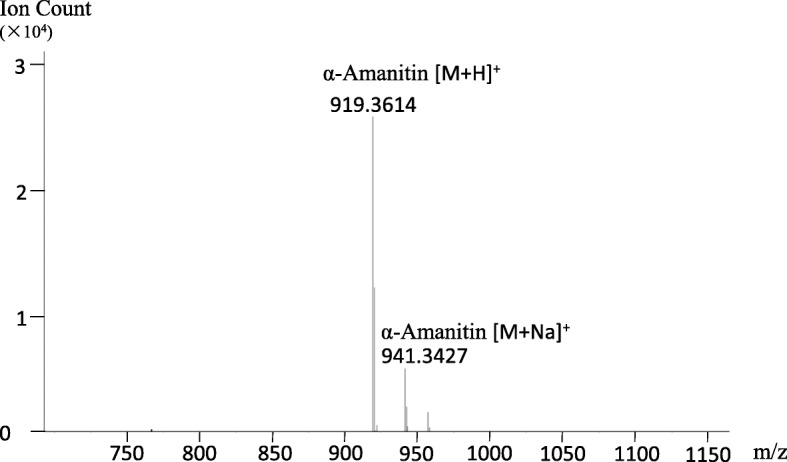
Mass spectrogram of α-amanitin produced in *Lepiota venenata*

Only one cyclic peptide, α-amanitin, was found in the extract (Fig. 2). α-Amanitin has a formula of C_39_H_54_N_10_O_14_S with a monoisotopic mass of 918.3541. The calculated mass of the [M + H^+^] ion is 919.3620, and the measured mass (918.3614) was within 10 ppm. The formula of β-amanitin is C_39_H_53_N_9_O_15_S and its calculated mass of the [M + H^+^] ion is 920.3620, which was not detected in the extract. The LC analyses detected a peak of 4.76 min, presenting the expected mass of protonized α-amanitin, and no beta-amanitin was found throughout the elution range (0–18 min). The masses of two other major toxins, phalloidin and phallacidin, were also applied in the scan, and no corresponding masses were identified. The result indicated that only one toxin, α-amanitin, was produced in *L. venenata*, which corresponded precisely with the genotype, i.e., the mushroom possesses only one MSDIN and it codes for α-amanitin. This result showed a different toxin profile compared to previous studies in other *Lepiota* species. Usually lethal *Lepiota* contains more than one toxin, for example, *L. brunneoincarnata* has a certain amount of both α-amanitin and β-amanitin [27], and α- and γ-amanitins are present in *L. josserandii* [6].

### Predicted structures of *LvAMA1* and *LvPOPB* genes

**Fig. 3:**
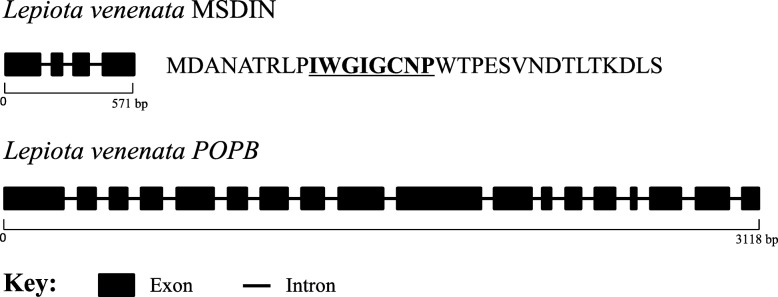
*POPB* and MSDIN structures of *Lepiota venenata*

The gene structures of *LvAMA1* and *LvPOPB* were predicted on Splign website (Fig. 3). The *LvAMA1* gene contained four exons and three introns. The coding sequence spanned the first intron, consisting of 99 bp. The *LvPOPB* gene was composed of 18 exons and 17 introns. The LvAMA1 protein had 32 amino acids, and the LvPOPB had 731 amino acids. Over all, the *LvPOPB* is very similar to other know *POPB* genes, in structure and in sequence.

### Phylogenetic analysis of *POP*

**Fig. 4:**
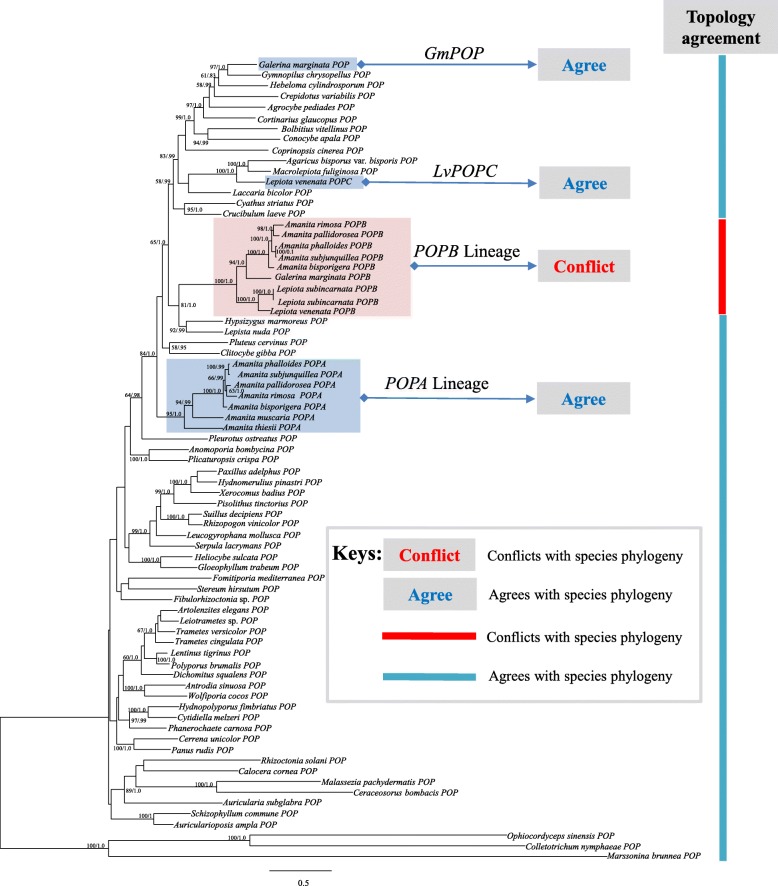
Phylogeny of macrofungi inferred from maximum likelihood (ML) analysis based on coding sequences of *POP* gene (Maximum likelihood bootstraps over 50% and Bayesian posterior probabilities over 0.90 are shown on the branches)

**Fig. 5:**
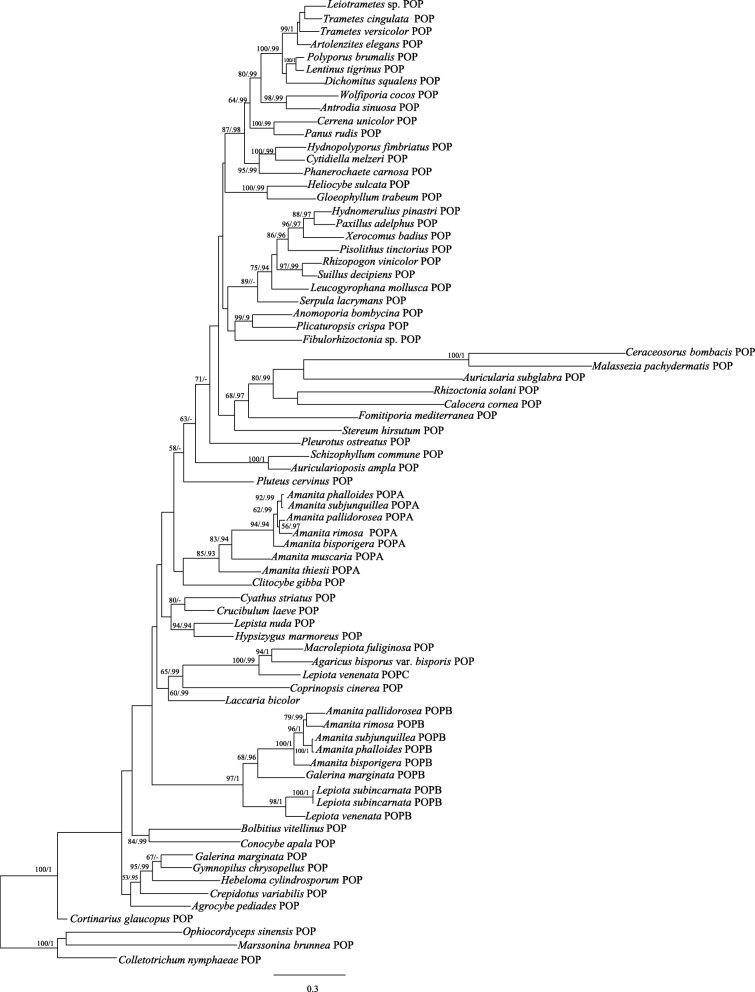
Phylogeny of macrofungi inferred from maximum likelihood (ML) analysis based on amino acid sequences of *POP* gene (Maximum likelihood bootstraps over 50% and Bayesian posterior probabilities over 0.90 are shown on the branches)

*POP* phylogenetic tree was constructed with three outgroups (*Ophiocordyceps sinensis*, *Marssonina brunnea* and *Colletotrichum nymphaeae*). As shown in Figs. 4 and 5, whether from the nucleotide or the amino acid phylogenetic tree, *POPB* genes were clustered together forming a highly monophyletic clade with strong support. However, *POPA* distribution was more complicated: *POPA*s of *Amanita* clustered together as expected, while that of *G. marginata* was separated from them nesting in a group containing *Gymnopilus chrysopellus* and *Cortinarius glaucopus*. “*POPA”*, referred as “generic *POP*” from now on, is considered as a house-keeping gene present in most basidiomycetes, which reflects the widely accepted phylogeny of the species included in the analyses [28–30]. Based on the result, “*POPA*” was used later in this report to refer to only *Amanita POPA*s.

**Fig. 6:**
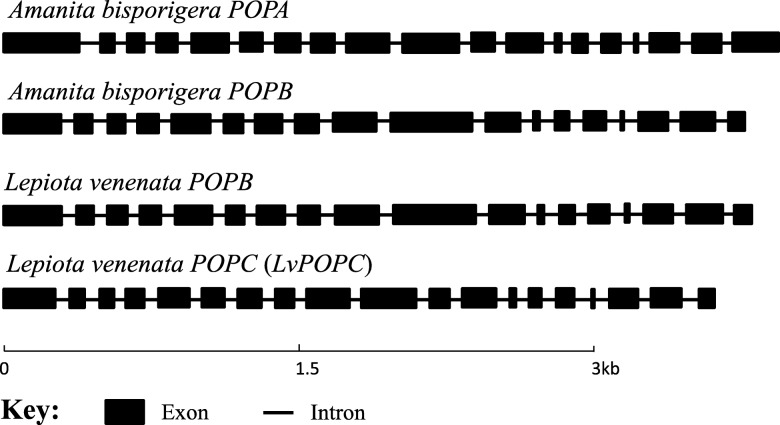
Structures of *POPB* from *Lepiota venenata* and *Amanita bisporigera*

A new *POP* gene was found in the genome of *L. venenata*, but surprisingly, it was not closely related to either *POPB* or *POPA* genes. As shown in Figs. 4 and 5, it was clustered with the *POP* analogs of *Laccaria bicolor* and *Macrolepiota fuliginosa*. We named it *LvPOPC* for reference purposes. The DNA and CDS sequences of *LvPOPC* were obtained through cloning and the detailed structure was shown in Fig. 6, which was compared with exemplary *POPA* and *POPB* from *A. bisporigera*. Both *POPA* of *A. bisporigera* (*AbPOPA*) and *LvPOPC* contain 19 exons and 18 introns. And *POPB* of *A. bisporigera* (*AbPOPB*) contains 18 exons and 17 introns. The length of *LvPOPC* sequence was the shortest while *AbPOPA* was the longest. One of the most significant differences was that the last exon length of *AbPOPA* was roughly twice the size of those in *AbPOPB* and *LvPOPC*. On the other hand, the pattern of exon-intron structure of *LvPOPC* was similar to that of *AbPOPA* and other *POPA*s, with 19 exons and 18 introns in similar arrangement, but significantly shorter. On the level of sequence similarity, *LvPOPC* was more divergent from both *POPA* and *POPB*, as it showed significantly lower identity during BLAST search. This explains why *LvPOPC* did not cluster with either *POPA* or *POPB*.

According to the nucleotide phylogenetic tree of *POP*s (Fig. 4), the taxa shaded in blue indicated the positions of generic *POP*s (*POPA*s, *GmPOP* and *LvPOPC*) in the three genera that produce α-amanitin. These phylogenetic positions were consistent with the typical species tree for Agaricales constructed by combined *rpb1*, *rpb1*-intron2, *rpb2*, 18S, 25S and 5.8S nucleotide sequences [30]. The results showed that generic *POP*s conformed to the agaric phylogeny. The taxa marked in red were the monophyletic branch of *POPB* with strong supportive statistics (Maximum likelihood bootstraps: 100% and Bayesian posterior probabilities: 1). The blue bars on the right of the figure indicated that all the taxa included in the analysis were consistent with the species phylogeny, except the *POPB* lineage (red bar). The *POPB*s of three different families violated the species phylogenetic relationships and were clustered into one monophyletic branch. Previous studies have shown that evolutionary tree analysis can be used as a powerful method to assess whether genes occur through HGT [31–33]. If the evolution of one gene with high sequence similarity in different species does not conform to the phylogenetic relationship, the gene may have HGT [34–36]. Our results showed that *POPB*s from three agaric families had high similarities in sequence and structure, but their phylogeny did not conform to the species phylogeny.

**Fig. 7:**
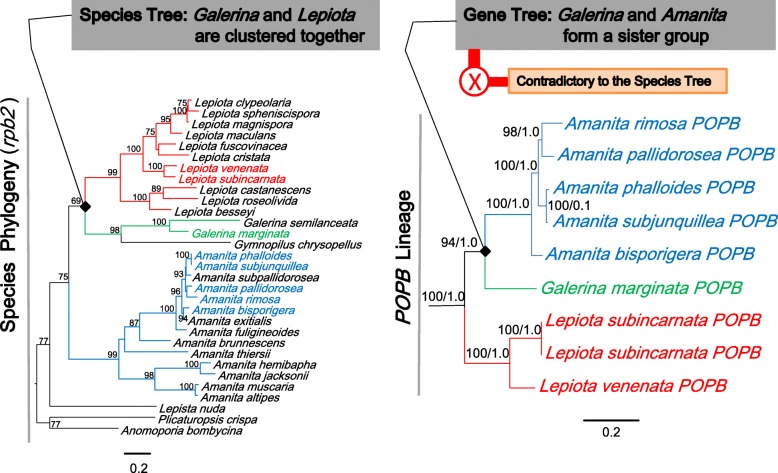
Comparison of *POPB* lineage with species tree. *Amanita* was indicated in Blue, *Lepiota* in red, and *Galerina* in green (Maximum likelihood bootstraps over 50% and Bayesian posterior probabilities over 0.90 are shown on the branches)

In order to further analyze the congruency between *POPB* lineage and the species phylogeny, we compared the lineage with a species tree based on *rpb2* (Fig. 7). In *POPB* lineage, the branches of *Galerina* and *Amanita* formed a sister group and the *Lepiota* species produced a monophyletic branch, clearly contradicting the species phylogeny indicated in the species tree, where *Galerina* and *Lepiota* were clustered together. Unlike our previous report, in this study the topology incongruency of the gene tree and the species tree is clear as described in the following, largely due to the newly found gene *LvPOPC*. *POPA* (from *Amanita* species), *GmPOP* and *LvPOPC* precisely reflected their phylogenetic positions in the widely accepted agaric phylogeny [30], and when massive gene loss is considered (i.e., species in between the genera removed from analysis), the collapsed phylogeny would be the order with *Amanita* at the base and *Galerina* at the terminal, conflicting with the order in *POPB* lineage, where *Lepiota* is basal and *Amanita* is terminal. The topology conflicts lent strong support to the hypothesis of horizontal gene transfer, while rejecting the hypothesis of massive gene loss.

### Topology test

To verify the robustness of the *POPB* branch, we used PAUP program to establish three hypothetical trees (see Methods), calculated their site-wise log-likelihoods, and then conducted approximately unbiased test with Consel. The results (Table 2) showed that, the approximately unbiased *p*-values (AU) and bootstrap probability (NP) of the three hypothetical trees were close to 0, while the value of best tree (the CDS *POP* phylogenetic tree) was 1. From the statistical point of view, the probability of occurrence of the best tree was 100%, while the three hypothetical trees were rejected. The evidence suggested that the *POPB* branch was robust and reliable.

**Table 2:** Approximately unbiased test on alternative *POP* trees

Rank	Best tree vs. Hypothetical trees	obs	AU	NP
1	Best tree	− 332.5	1.000	1.000
2	*POPB*s were monophyletic with *Amanita POPA*	332.5	3.00E-05	3.00E-06
3	*POPB*s were monophyletic with *Amanita POPA* and *Galerina POP*s	334.8	1.00E-86	9.00E-23
4	*POPB*s were monophyletic with *Galerina POP*s	903.6	2.00E-37	5.00E-15

Notes: *obs* observed log-likelihood difference

*AU* approximately unbiased *p*-values

*NP* bootstrap probability

### Gene tree and species tree

In order to evaluate evolutionary history of *POPB*, we established a species tree and a gene tree similar to those in our previous report [14]. The species tree faithfully reflected the phylogenetic relationship of *Amanita*, *Galerina* and *Lepiota*. For the gene tree, the branches of *POPA* and *POPB* were clustered with their own homologs, respectively. With Notung, the Divergence-Loss models (DL model) returned the following general statistics: Event Score = 38.5, Dups = 5, Losses = 31, and Numbers of optimal solutions = 1. The Divergence-Transfer-Loss (DTL model) produced: Event Score = 24.0, Dups = 0, Transfers = 5, and Losses = 9. The DTL Event score was significantly smaller than that of DL model. This indicated that the probability of DTL model with HGT in *POPB* was significantly higher than that in gene duplication and loss only assumption.

In addition, a *POP* and a MSDIN gene trees similar to those in our recent report [14] were constructed. These trees were analyzed by PAML software. The species tree represented the phylogeny of the housekeeping gene (*rpb2*), while the gene trees represented the evolutionary relationships of the *POP* and MSDIN genes. The results showed that there were significant differences in distances, synonymous rates (dS) and nonsynonymous rates (dN) among the three amanitin-producing genera. The distances of *rpb2* was significantly higher than those of *POPB* (2 ~ 6 times, Table 3) and MSDIN (up to 3 times, Table 3). The data in the table also showed that the dN/dS values of *POPB* were small, and the dS ratio for *A. phalloides* and *G. marginata* was about 1:7, while the dN ratio was close to 2:1. These results demonstrated that the distances of *POPB* and MSDIN genes were significantly shorter than those of *rpb2*, and therefore the evolution of *POPB* and MSDIN was not in accordance with vertical inheritance. These data are consistent with HGT scenario as significantly lower rates are expected when compared to house-keeping genes.

**Table 3:**
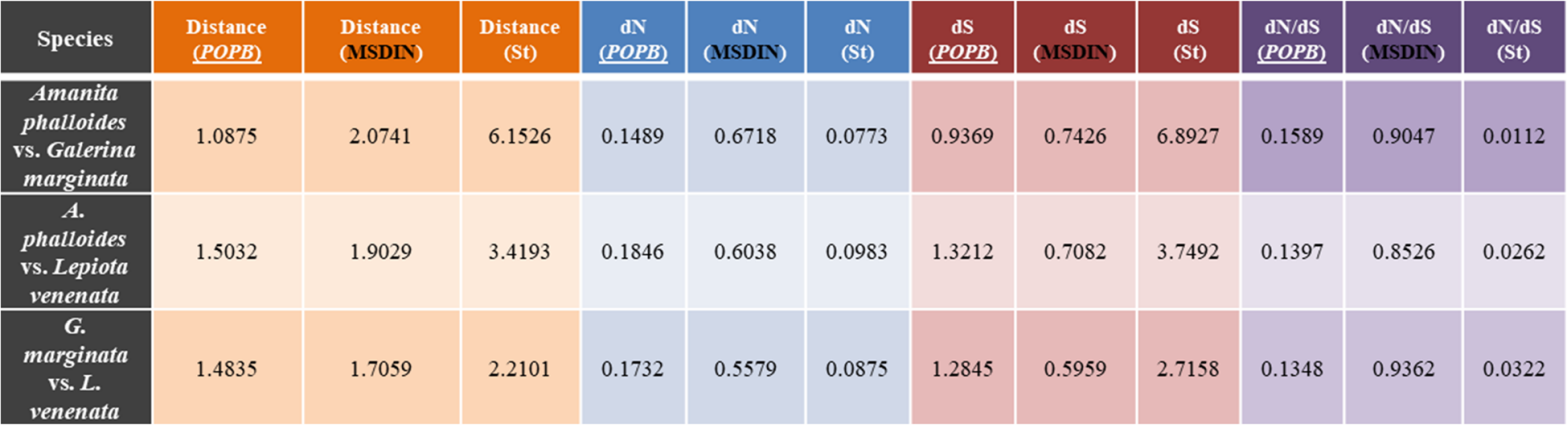
Likelihood ratio tests of hypotheses through comparison of implemented models among the three amanitin-producing genera

Note:

St: Species tree

*POPB*s were underlined

MSDINs were highlighted in black

## Discussion

### *Lepiota* genome

α-Amanitin, the major cyclic peptide toxin, is responsible for the vast majority (> 90%) of deadly mushroom poisonings worldwide [37]. The organisms containing this toxin are known to be distributed in the three disjunct agaric genera, *Amanita*, *Galerina* and *Lepiota* [4, 38–41]. Draft genomes are available for both *Amanita* and *Galerina*, but not for *Lepiota* due to the difficulty of obtaining high quality material. In China, there has been more than a few deadly poisoning cases caused by *Lepiota* in the past three years [25], allowing acquisition of samples suitable for genome sequencing. To the best of our knowledge, this is the first available genome of *Lepiota,* which now allows studies of the toxin biosynthesis on full spectrum, i.e., including all known α-amanitin-containing genera. Research on the mechanism of the toxin biosynthesis and toxin genes of lethal *Lepiota* species also has a practical significance for establishing prevention and control systems for the poisonings in the future. As an example, primers targeting the toxin genes in *Lepiota* have been developed for rapid PCR identification of these mushrooms by our group.

Most of the genomic statistics of *L. venenata* are within the ranges of those in related agaric genomes [14, 17, 20–23]. One aspect does stand out: the genomic GC content of the *L. venenata* genome, 49%, is the highest of the three genera. In addition, there is a clear distinction between the saprotrophic and symbiotic agarics. The GC content of symbiotic *Amanita* is the lowest, such as *A. phalloides* and *A. bisporigera*, at 42.2 and 43.2%, respectively [20]. The genomic GC content of the saprotrophic mushrooms is higher: it is 48% for *G. marginata* [42], close to that of *L. venenata* at 49%. Our other ongoing sequencing projects with additional *Amanita* and *Lepiota* species conform to this trend. GC content often correlates in negative relationship with amount of repetitive sequences. Our ongoing sequencing of *A. subjunquillea* and *A. pallidorosea* yielded 53.26 and 55.07% of the respective genomes as repetitive sequences. For *L. venenata* the percentage dropped to 26.81%. The significant differences between *L. venenata* and the two *Amanita* species are consistent with the negative correlation.

### Ribosomal nature of amanitin biosynthetic pathway

*Lepiota venenata* possesses both MSDIN and *POPB* genes, and the structures of these genes are consistent with those of their counterparts in both *Amanita* and *Galerina* [1, 14, 18]. Clearly, *L. venenata* uses the same ribosomal mechanism to produce α-amanitin as in *Amanita* [17, 20] and *Galerina* [18]. The genes involved in the pathway, homology, and exon-intron structures, clearly indicate these pathways in the three genera are related. At this point, it may be safe to conclude that, for all the α-amanitin-producing mushrooms, the same ribosomal biosynthetic pathway based on MSDIN and *POPB* genes, is universally used for amanitin production.

### *LvPOPC* and evolution of *POPB* genes

*LvPOPC* is critical for the phylogenetic reconstruction of *POP* history, and it was the reason that our previous report [14] was not able to fully rule out massive gene loss by comparing the gene tree with the species tree. Since phylogenetic conflict is still considered as the most powerful method to investigate HGT [43, 44], we repeated the analysis with the addition of *LvPOPC*. As shown in Figs. 4 and 5, the *POPB* lineage was the only clade that conflicted with the species phylogeny (red bar in Fig. 4), and the topology conflict between the gene tree and the species tree became indisputable even when massive gene loss was considered. This result has lent strong support to the HGT hypothesis of *POPB*.

The reason we (and our colleagues) missed *LvPOPC* in the beginning was that it has low homology to the query *POPA* and *POPB* sequences*.* After the gene was cloned and structure determined, we realized that it is in a way similar to *POPA*, with similar exon-intron pattern, although shorter in length. The high divergence in the sequence may be due to its separation from other *POP*s long time ago.

### MSDIN genes

*Lepiota venenata* only possesses one MSDIN sequence in the draft genome, which is consistent with the toxin profile of this mushroom (Fig. 2). The scenario is similar to that in *G. marginata* [18], and both of the mushrooms are saprotrophic agarics. Furthermore, two MSDIN sequences found in the genome of *L. brunneoincarnata* also encode α-amanitin [14]. In *Amanita*, subsets of MSDIN genes oftentimes reach the range of 20 to 30 [3, 17, 20, 45]. MSDIN genes in *Amanita* are conserved in the leader peptide region, and they mostly start with the amino acid string of MSDIN. In contrast, the leader peptide in *Galerina* begins with MFDTN, and our ongoing sequencing of *G. sulciceps* confirms this (data not shown). In *Lepiota*, the second amino acid in the leader peptide region is missing, starting with M-DAN. These sequences are clearly related, and phylogenetic reconstruction of these genes showed that, on genus level they cluster by their taxonomic distribution, but within a genus they group by function, i.e., MSDIN sequences encoding same cyclic peptides group together (data not shown). This result is consistent with our recent report [14].

### HGT scenarios: Transposon and gene cluster

Unlike vertical inheritance, HGT is transfer of genetic material between unicellular or multicellular organisms, rather than from parents to offspring [44, 46]. HGT plays an important role in the evolution of many organisms [47, 48]. Transposons are pieces of DNA that can be moved in a genome [49, 50]. Horizontal transfer is an important way for transposons to avoid extinction in the host genome due to purifying selection, genetic drift or mutation inactivation [51–53], and it is considered to be an important driving force for genome mutation and biological transformation [54]. Transposons are divided into two categories, which can be described as copy-paste (class I) or cut-and-paste (class II) [55, 56]. In *L. venenata*, transposons accounted for 26.81% of the whole genome and were distributed on every contig. In addition, we analyzed and counted the numbers of transposons within 50 Kb upstream and downstream of *LvPOPB* and *LvAMA1*. There are 5 transposons in the vicinity of *POPB*, which belong to Class I (retrotransposons). Near *LvAMA1*, there are 4 transposons, two of which belong to Class I, and the other two belong to Class II (DNA transposons). Although the mechanisms of HGT are not fully understood, transposon may play a role in the event, and further research is needed in the direction.

Supernumerary chromosome transfers can also be explained by interspecific mating rather than HGT [57, 58], and similarly, horizontal chromosome transfer (HCT) can be the underlining cause as well [59, 60]. These hypotheses can be readily tested via comparative genomic approaches, and our analyses using Symap were negative on both assumptions.

In the *L. venenata* genome, *POPB* gene is located far from the only MSDIN, *LvAMA1*, with at least 100 predicted genes in between. The result indicates it may be a case of single gene transfer instead of gene cluster transfer. Our other ongoing sequencing of *L. brunneoincarnata* returned the same result (at least 200 Kb apart, with hundreds of predicted genes in between).

### Evolutionary history of the biosynthetic pathway

Collectively, the *L. venenata* genome offered new evidence that the key toxin-biosynthetic gene, *POPB*, is acquired through HGT. The genetic distances of MSDIN genes also support that MSDIN underwent the same process. *POPB* catalyzes the cyclization of the precursor peptide and is at the very center of the pathway. Therefore the HGT nature of this gene would likely apply to the entire pathway. Multiple lines of evidence support the HGT hypothesis. First, significant similarities are found in *POPB* and MSDIN gene sequences and structures across three distant families, indicating these genes are related and did not arise independently. From the phylogenetic and topology analyses, the *POPB* branch is robust and does not conform to the species phylogeny. The genetic distances of both *POPB* and MSDIN genes are considerably shorter than those of *rbp2*, consistent with the HGT scenario. The detailed comparison of *POPB* lineage to the species phylogeny illustrated that the conflict is clear, ruling out massive gene loss.

## Conclusions

The genome of *L. venenata* illustrated that a ribosomal mechanism is the underlining mechanism of its amanitin biosynthesis. The toxin genes, *LvAMA1* and *LvPOPB*, were far apart in the genome, not forming a gene cluster. The evidence suggested that, in the three disjunct genera, *Amanita*, *Lepiota* and *Galerina*, these toxin genes were acquired through HGT mechanism.

## Methods

### Biological materials

The mushroom fruiting bodies used in this study was *L. venenata*, collected in Jingzhou City, Hubei Province of central China. The fungal materials were identified by the second and the second-to-last authors who published the new species in Cai et al [25]. Specimens were deposited in the Herbarium of Cryptogams, Kunming Institute of Botany, Chinese Academy of Sciences (holotype, HKAS 101874), and Mycological Herbarium of Hunan Normal University (isotype, MHHNU 31031), respectively. The fruiting bodies were collected and wrapped in tin foil, immediately put on dry ice, and subsequently stored in − 80 °C before use.

### Genome sequencing and assembly

The sequencing platform for the genome of *L. venenata* used PacBio Sequel at NextOmics Biosciences, Wuhan, China. Sequencing and assembly were carried out using the company’s standardized pipeline briefly described as the following. High quality DNA was extracted and examined for high molecular weight suitable for 20-Kb library construction. The genomic DNA was then randomly interrupted with Covaris g-TUBE. Large fragment of DNA was enriched and purified by magnetic beads. Then, the fragmented DNA was repaired. At the ends of DNA fragments, the stem circular sequencing joint was connected, and unconnected fragments removed by exonuclease. A 20-Kb library was constructed using a PacBio template prep kit and analyzed by Agilent 2100 Bioanalyzer for quality control. After the completion of the library, the DNA template and enzyme complex were transferred to the Sequel sequencer for real-time single molecule sequencing. Illumina HiSeq X10 platform was used for nucleotide level correction, based on a 350 bp library constructed, and the company’s a standard method was applied.

### Sequences of MSDIN and *POP* genes

Nucleotide sequences of MSDIN and *POP* genes from *L. venenata* genome were obtained by BLAST (NCBI BLAST^+^ 2.4.0). Query MSDIN and *POPB* sequences came from *A. bisporigera* and *G. marginata*, which are well characterized by our previous molecular and biochemical approaches [1, 17, 18]. Comparison was also done with related sequences from *A. phalloides* [20]. After alignment of MSDIN and *POP* gene sequences, the introns and exon positions were predicted by MegAlign v7.1.0 following our previous method [14]. After the introns were removed, predicted MSDIN and *POPB* CDS sequences of *L. venenata* were obtained and named *LvAMA1* and *LvPOPB*, respectively. One additional sequence with weak homology to both *POPA* and *POPB* was identified and denoted as *LvPOPC*. This gene was further cloned as described below.

### Cloning and structure prediction of *LvPOPC* and *LvAMA1*

CDS sequences of *LvPOPC* and *LvAMA1* were obtained by reverse transcription PCR (RT-PCR) using primers based on the genomic data. As BLAST result indicated, one stretch of genomic DNA sequence has homology (although weak) to both *POPA* and *POPB*. This particular DNA sequence was selected for primer design aiming to cover the full coding region, using those in *POPA* and *POPB* as the references. For the *LvPOPC*, the forward primer was 5′-CCCGGGTTGTAGTGGTGTAAGG-3′, and the reverse primer was 5′-ACATATTATCTCCCTGCTTTCACC-3′. For the *LvAMA1*, the forward primer was 5′-TCTCCAGGCCTCATTCACATTACC-3′ and the reverse primer was 5′-TGCCAGACACGGAACAAATACATC-3′. The reactions were carried out under standard conditions. The structures of the genes were illustrated using the CDS and gDNA sequences on Splign website (https://www.ncbi.nlm.nih.gov/sutils/splign/splign.cgi? The textpage = online&level = form).

### Visualization of the genome and toxin genes

In this study, genome visualization software Circos [61] was chosen to map the genome and toxin genes. Python scripts for obtaining GC content and GC skew were generated. Genome annotation files were processed mainly through Excel, and the resulted track files were used for building information tracks in Biopython environment. The tracks were loaded into Circos to produce a genome overview in Perl environment. Coordinates of *LvPOPB* and *LvAMA1* genes were loaded as a track to show their precise genomic locations.

### Analysis of toxin by LC-MS

To verify if the genomic potential for cyclic peptide in *L. venenata* is consistent with the actual toxin phenotype, a liquid chromatography-mass spectrometry (LC-MS) method was applied on Aglient 6530 series system (Agilent Technologies, Palo Alto, CA, USA).

Toxins were extracted from fruiting bodies, using methanol: water: 0. 01 M hydrochloric acid (5: 4: 1) as the extraction buffer. Then, 0.06 g dried material was weighed and ground to fine powder in liquid nitrogen, 2 ml buffer were added, and the suspension transferred to 1.5 ml centrifuge tubes. The tubes sit at room temperature for 30 min, followed by centrifugation (12,000 rpm) for 3 min. Finally, the supernatant was boiled, centrifuged again, and supernatant transferred to fresh centrifuge tubes.

### Phylogenetic analyses of *POP* genes

Coding sequences (CDS) and amino acid sequences of selected *POP* genes for the phylogenetic analyses were obtained from NCBI (https://www.ncbi.nlm.nih.gov/) and JGI (http://genome.jgi.doe.gov/programs/fungi/index.jsf) (Additional file 3). Those sequences were aligned by MAFFT v7.304b [62] with default settings, and then manually adjusted with BioEdit [63] (Additional files 4 and 5). The CDS sequences and amino acid sequences of *POP* include 75 taxa, of which three are chosen as outgroups. For the amino acid alignment, the best model LG + G was detected by ProTest 3.4.2 [64] under Akaike Information Criterion (AIC). For the nucleotide alignment, GTR + I + G was selected as the best model for the CDSs of *POP* genes, using MrModeltest v2.3 [65] under AIC. Maximum Likelihood analyses and bootstrapping (1000 replicates) were performed using RAxML v7 [66]. For Bayesian inference analyses, MrBayes v3.2.6 [67] was used under the optimal substitution model calculated from MrModeltest. The posterior distributions of parameters were obtained using Markov chain Monte Carlo (MCMC) analysis for 20 million generations. Chain convergence was determined when the stopval equals to 0.01. The sampled trees were summarized after omitting the first 25% of trees as burn-in.

### Topology test

In order to test whether the *POPB* branch in the *POP* phylogenetic tree is reliable, hypothetical trees were built to compare with the best tree (the CDS *POP* phylogenetic tree). Three different hypothetical trees were built by PAUP v4.0b10, and their site-wise log-likelihoods obtained. These hypotheses were (1) *POPB*s and *Amanita POPA* were monophyletic, (2) *POPB*s, *Amanita POPA* and *Galerina POP*s were monophyletic, and (3) *POPB*s were monophyletic with *Galerina POP*s. Site-wise log-likelihoods for the above hypothetical threes were generated and transferred into Consel v0.1i to perform approximately unbiased tests [68].

### Gene tree vs. species tree

The *POP* gene and species trees were constructed from 20 taxa and 31 taxa, respectively. These taxa included representative species from *Amanita*, *Galerina* and *Lepiota*. For species tree, the 31 taxa (Additional file 6) contain all the 20 taxa in gene tree (Additional file 7). The *rpb2* sequences [14] were obtained from our custom genomes, GenBank and JGI genomes (blastp) using *A. subpallidorosea rpb2* (KP691703) as the query. Both gene tree and species tree were constructed by RAxML v7 and MrBayes v3.2.6, and the best model was GTR + I + G. *Plicaturopsis crispa* and *Anomoporia bombycina* were chosen as the outgroups. The resultant gene and species trees were analyzed by Notung 2.9 [69] with Divergence-Loss (DL) and Divergence-Transfer-Loss (DTL) models under default settings. In addition, in order to further study the relationship between the *POP* gene and species trees, we used codeml program in PMAL v4.9 [70] for post-phylogenetic analysis, including distance and divergence rate calculations.

## Additional files

Additional file 1: Nucleotide sequence of *LvAMA1*. (DOCX 15 kb)

Additional file 2: Nucleotide sequence of *LvPOPB. (DOC 30 kb)*

Additional file 3: Accession numbers of prolyl oligopeptidase gene and amino acid sequences included in the phylogenetic study. (DOCX 25 kb)

Additional file 4: Alignment of *POP* coding sequences from 75 taxa. (PHY 376 kb)

Additional file 5: Alignment of *POP* amino acid sequences from 75 taxa. (PHY 105 kb)

Additional file 6: Alignment of *rpb2* coding sequences in species tree. (PHY 29 kb)

Additional file 7: Alignment of *POP* coding sequences in gene tree. (PHY 62 kb)

## Abbreviations

LC: Liquid chromatography
MS: Mass spectrometry
POP: Prolyl oligopeptidase

## References

[1] Luo H, Hallen-Adams HE, Scott-Craig JS, Walton JD (2010). Colocalization of amanitin and a candidate toxin-processing prolyl oligopeptidase in *Amanita* basidiocarps. Eukaryot Cell.

[2] Deng WQ, Li TH, Xi PG, Gan LX, Xiao ZD, Jiang ZD (2011). Peptide toxin components of *Amanita exitialis* basidiocarps. Mycologia..

[3] Li P, Deng WQ, Li TH, Song B, Shen YH (2013). Illumina-based de novo transcriptome sequencing and analysis of *Amanita exitialis* basidiocarps. Gene..

[4] Haines JH, Lichstein E, Glickerman D (1986). A fatal poisoning from an amatoxin containing *Lepiota*. Mycopathologia..

[5] Mottram AR, Lazio MP, Bryant SM (2010). *Lepiota subincarnata* J.E. Lange induced fulminant hepatic failure presenting with pancreatitis. J Med Toxicol.

[6] Sgambelluri RM, Epis S, Sassera D, Luo H, Angelos ER, Walton JD (2014). Profiling of amatoxins and phallotoxins in the genus *Lepiota* by liquid chromatography combined with UV absorbance and mass spectrometry. Toxins..

[7] Singer R (1986). The Agaricales in modern taxonomy.

[8] Yang ZL (1997). Die *Amanita*-Arten von Südwestchina. Bibl Mycol.

[9] Moncalvo JM, Vilgalys R, Redhead SA, Johnson JE, James TY, Catherine Aime M (2002). One hundred and seventeen clades of euagarics. Mol Phylogenet Evol.

[10] Justo A, Morgenstern I, Hallen-Adams HE, Hibbett DS (2010). Convergent evolution of sequestrate forms in *Amanita* under Mediterranean climate conditions. Mycologia..

[11] Cui YY, Cai Q, Tang LP, Liu JW, Yang ZL (2018). The family Amanitaceae: molecular phylogeny, higher-rank taxonomy and the species in China. Fungal Divers.

[12] Gulden G, Stensrud O, Shalchian-Tabrizi K, Kauserud H (2005). *Galerina* Earle: a polyphyletic genus in the consortium of dark-spored agarics. Mycologia..

[13] Vellinga EC (2003). Phylogeny of *Lepiota*, (Agaricaceae) evidence from nrITS and nrLSU sequences. Mycol Prog.

[14] Luo H, Cai Q, Lüli YJ, Li X, Sinha R, Hallen HE (2018). The MSDIN family in amanitin-producing mushrooms and evolution of the prolyl oligopeptidase genes. IMA Fungus..

[15] Walton JD (2018). The cyclic peptide toxins of *Amanita* and other poisonous mushrooms.

[16] Chen ZH, Yang ZL, Bau T, Li TH (2016). Poisonous mushrooms: recognition and poisoning treatment.

[17] Hallen HE, Luo H, Scott-Craig JS, Walton JD (2007). Gene family encoding the major toxins of lethal *Amanita* mushrooms. Proc Natl Acad Sci U S A.

[18] Luo H, Hallen-Adams HE, Scott-Craig JS, Walton JD (2012). Ribosomal biosynthesis of α-amanitin in *Galerina marginata*. Fungal Genet Biol.

[19] Luo H, Hong SY, Sgambelluri RM, Angelos E, Li X, Walton JD (2014). Peptide macrocyclization catalyzed by a prolyl oligopeptidase involved in α-amanitin biosynthesis. Chem Biol.

[20] Pulman JA, Childs KL, Sgambelluri RM, Walton JD (2016). Expansion and diversification of the MSDIN family of cyclic peptide genes in the poisonous agarics *Amanita phalloides* and *A. bisporigera*. BMC Genomics.

[21] Kohler A, Kuo A, Nagy LG, Morin E, Barry KW, Buscot F (2015). Convergent losses of decay mechanisms and rapid turnover of symbiosis genes in mycorrhizal mutualists. Nat Genet.

[22] van der Nest Ma BLA, Crouch JA, Demers JE, de Beer ZW, De Vos L (2014). Draft genomes of *Amanita jacksonii*, *Ceratocystis albifundus*, *Fusarium circinatum*, *Huntiella omanensis*, *Leptographium procerum*, *Rutstroemia sydowiana*, and *Sclerotinia echinophila*. IMA Fungus.

[23] Hess J, Skrede I, Wolfe BE, Labutti K, Ohm RA, Grigoriev IV (2014). Transposable element dynamics among asymbiotic and ectomycorrhizal *Amanita* fungi. Genome Biol Evo.

[24] Wieland T (1986). Peptides of poisonous *Amanita* mushrooms.

[25] Cai Q, Chen ZH, He ZM, Luo H, Yang ZL (2018). *Lepiota venenata*, a new species related to toxic mushroom in China. J Fungal Res.

[26] Luo H, Hallen-Adams HE, Walton JD (2009). Processing of the phalloidin proprotein by prolyl oligopeptidase from the mushroom *Conocybe albipes*. J Biol Chem.

[27] Yilmaz I, Bakirci S, Akata I, Bayram R, Kaya E (2015). Toxin content and toxicological significance in different tissues and development stages of *Lepiota brunneoincarnata* mushroom. Toxin Rev.

[28] Hibbett DS (2006). A phylogenetic overview of the Agaricomycotina. Mycologia..

[29] Matheny PB, Curtis JM, Hofstetter V, Aime MC, Moncalvo JM, Ge ZW (2006). Major clades of Agaricales: a multilocus phylogenetic overview. Mycologia..

[30] Matheny PB, Wang Z, Binder M, Curtis JM, Lim YW, Nilsson RH (2007). Contributions of *rpb2* and *tef1* to the phylogeny of mushrooms and allies (Basidiomycota, Fungi). Mol Phylogenet Evol.

[31] Doolittle WF (1999). Phylogenetic classification and the universal tree. Science..

[32] Gogarten JP, Doolittle WF, Lawrence JG (2002). Prokaryotic evolution in light of gene transfer. Mol Biol Evo.

[33] Bapteste E, Susko E, Leigh J, Macleod D, Charlebois RL, Doolittle WF. Do orthologous gene phylogenies really support tree-thinking? BMC Evol Biol 2005;5:33. 10.1186/1471-2148-5-33 PMC1156881 15913459

[34] Li ZJ, Li HQ, Diao XM (2008). Methods for the identification of horizontal gene transfer (HGT) events and progress in related fields. Yi Chuan.

[35] Jain R, Rivera MC, Lake JA (1999). Horizontal gene transfer among genomes: the complexity hypothesis. Proc Natl Acad Sci U S A.

[36] Koonin EV, Makarova KS, Aravind L (2001). Horizontal gene transfer in prokaryotes: quantification and classification. Annu Rev Microbiol.

[37] Bresinsky A, Besl H (1990). A colour atlas of poisonous fungi: a handbook for pharmacists, doctors and biologists.

[38] Vetter J (1998). Toxins of *Amanita phalloides*. Toxicon..

[39] Enjalbert F, Rapior S, Nouguier-soulé J, Guillon S, Amouroux N, Cabot C (2002). Treatment of amatoxin poisoning: 20-year retrospective analysis. J Toxicol Clin Toxicol.

[40] Enjalbert F, Cassanas G, Rapior S, Renault C, Chaumont JP (2004). Amatoxins in wood-rotting *Galerina marginata*. Mycologia..

[41] Zhang P, Chen Z, Hu J, Wei B, Zhang Z, Hu W (2005). Production and characterization of amanitin toxins from a pure culture of *Amanita exitialis*. FEMS Microbiol Lett.

[42] Riley R, Salamov AA, Brown DW, Nagy LG, Floudas D, Held BW (2014). Extensive sampling of basidiomycete genomes demonstrates inadequacy of the white-rot/brown-rot paradigm for wood decay fungi. Proc Natl Acad Sci U S A.

[43] Andersson JO (2005). Lateral gene transfer in eukaryotes. Cell Mol Life Sci.

[44] Keeling PJ, Palmer JD (2008). Horizontal gene transfer in eukaryotic evolution. Nat Rev Genet.

[45] Li P, Deng W, Li T (2014). The molecular diversity of toxin gene families in lethal *Amanita* mushrooms. Toxicon..

[46] Ochman H, Lawrence JG, Groisman EA (2000). Lateral gene transfer and the nature of bacterial innovation. Nature..

[47] Gyles C, Boerlin P (2014). Horizontally transferred genetic elements and their role in pathogenesis of bacterial disease. Vet Pathol.

[48] Vaux F, Trewick SA, Morgan-Richards M (2016). Speciation through the looking-glass. Biol J Linn Soc Lond.

[49] Mcclintock B (1950). The origin and behavior of mutable loci in maize. Proc Natl Acad Sci U S A.

[50] Feschotte C (2008). Transposable elements and the evolution of regulatory networks. Nature Rev Genet.

[51] Daniels SB, Peterson KR, Strausbaugh LD, Kidwell MG, Chovnick A (1990). Evidence for horizontal transmission of the *P* transposable element between *Drosophila* species. Genetics..

[52] Hartl DL, Lohe AR, Lozovskaya ER (1997). Modern thoughts on an ancyent *marinere*: function, evolution, regulation. Annu Rev Genet.

[53] Robertson HM, Craig N, Craigie R, Gellert M, Lambowitz A (2002). Evolution of DNA transposons in eukaryotes. Mobile DNA II.

[54] Schaack S, Gilbert C, Feschotte C (2010). Promiscuous DNA: horizontal transfer of transposable elements and why it matters for eukaryotic evolution. Trends Ecol Evol.

[55] Feschotte C, Pritham EJ (2007). DNA transposons and the evolution of eukaryotic genomes. Annu Rev Genet.

[56] Kapitonov VV, Jurka J (2008). A universal classification of eukaryotic transposable elements implemented in Repbase. Nat Rev Genet..

[57] Masel AM, He C, Poplawski AM, Irwin JAG, Manners JM (1996). Molecular evidence for chromosome transfer between biotypes of *Colletotrichum gloeosporioides*. Mol Plant-Microbe Interact.

[58] Mehrabi R, Bahkali AH, Abd-Elsalam KA, Moslem M, Ben M'Barek S, Gohari AM (2011). Horizontal gene and chromosome transfer in plant pathogenic fungi affecting host range. FEMS Microbiol Rev.

[59] Akagi Y, Akamatsu H, Otani H, Kodama M (2009). Horizontal chromosome transfer, a mechanism for the evolution and differentiation of a plant-pathogenic fungus. Eukaryot Cell.

[60] Ma LJ, van der Does HC, Borkovich KA, Coleman JJ, Daboussi MJ, Pietro AD (2010). Comparative genomics reveals mobile pathogenicity chromosomes in *Fusarium*. Nature..

[61] Krzywinski M, Schein J, Birol I (2009). Circos: an information aesthetic for comparative genomics. Genome Res.

[62] Katoh K, Standley DM (2016). A simple method to control over-alignment in the MAFFT multiple sequence alignment program. Bioinformatics..

[63] Hall TA (1999). BioEdit: a user-friendly biological sequence alignment editor and analysis program for windows 95/98/NT. Nucleic Acids Sym Ser.

[64] Darriba D, Taboada GL, Doallo R, Posada D (2011). ProtTest 3: fast selection of best-fit models of protein evolution. Bioinformatics..

[65] Nylander JAA. MrModeltest v2.2 Uppsala: evolutionary biology Centre, Uppsala University. 2004.

[66] Stamatakis A (2006). RAxML-VI-HPC: maximum likelihood-based phylogenetic analyses with thousands of taxa and mixed models. Bioinformatics..

[67] Ronquist F, Huelsenbeck JP (2003). MrBayes 3: Bayesian phylogenetic inference under mixed models. Bioinformatics..

[68] Shimodaira H, Hasegawa M (2001). CONSEL: for assessing the confidence of phylogenetic tree selection. Bioinformatics..

[69] Chen K, Durand D, Farach-Colton M (2000). NOTUNG: a program for dating gene duplications and optimizing gene family trees. J Comput Biol.

[70] Yang Z (2007). PAML 4: phylogenetic analysis by maximum likelihood. Mol Biol Evol.

