# Supplementary material for: Genome of lethal Lepiota venenata and insights into the evolution of toxin-biosynthetic genes
Source: BMC Genomics. 2019 Mar 8;20:198. doi: 10.1186/s12864-019-5575-7 (PMC6408872; doi:10.1186/s12864-019-5575-7)
Supplement: Supplementary file 1 — Nucleotide sequence of LvAMA1. (DOCX 15 kb) [file 12864_2019_5575_MOESM1_ESM.docx]

Genomic DNA and amino acid sequences of *LvAMA1* (introns underlined, start and stop codons in red):

1: DNA sequence

GAATCTCCAGGCCTCATTCACATTACCTACACTCGAACCCATCACTCTTACTCTTTGATTACAATGGACGCCAACGCCACCCGTCTCCCAATCTGGGGTATTGGTTGCAACCCATGGACTCCCGAGAGTGTCAACGACACTCTGACTAAGGAGTGAGCTCAACATTTGCTGAAGCCCAGATCCGACGCACTTATTGTTCCCCATCAGCCTTTCCTAAATATCCTGCCCCTAAGTCCGCCATGGCCCGAAGGTCGTCCATCATCTTGCTCTTCTGTTCGACCCCAGTACTAACAAATGCATAGTGGTGGACGTGGGCAGGATGTGGCTTGTCGACGGTATCGGGCTTGGAGCTTTAAGCCTGGATCGAAGGTCAGACACTGCGTCGCGCTGCAATTATGCGGGAAATGCTGACTTGGCTTCTACTTTTTGTCAGACTATCACATTCTTTCTCGTTGAATTCTAAATCAGTTTGACTCAGTAAATATATAAATCCATGTTGTTTATCTGCGATGTTATGTTGAATGGATGTATTTGTTCCGTGTCTGGCAACCTCCACAAAATGAGCTGGGTA

2: Amino acid sequence

MDANATRLPIWGIGCNPWTPESVNDTLTKDLS
