# Supplementary material for: Genome of lethal Lepiota venenata and insights into the evolution of toxin-biosynthetic genes
Source: BMC Genomics. 2019 Mar 8;20:198. doi: 10.1186/s12864-019-5575-7 (PMC6408872; doi:10.1186/s12864-019-5575-7)
Supplement: Supplementary file 2 — Nucleotide sequence of LvPOPB. (DOC 30 kb) [file 12864_2019_5575_MOESM2_ESM.doc]

Genomic DNA and amino acid sequences of *LvPOPB* (Underline indicates introns):

1: DNA sequence

ATGCCTCCTACGCTGTGGACTACCGCAAAATATCCCTCTGCTCGCCGTTCTGATCACGTCGATGTCTATAAGAGCGCGCTGAAGGGCGAAGTACACATACCGGACCCGTACCAATGGCTCGAGGGGAACTCCGAGGAGGTGGACGACTGGATGACGGCCCAAGCCGCCTTCACACGTGCCTACCTCGACCAAAACCAAGGCAGCCACGAGCTTGAGGACAAGTTCCGTGCCAACCTGAATTATGCAAAGGTGAACCATTCGCCTTTCTCTGGTTCTAGTCGATATGCTTATCGTGTGCTGCGGCAGTTCTCCGCGCCAACTTTACTTGATGATGGACATTGGTACTGGTTTTACAATAGTGGTCTACAATCACAGTCTGGTGCGCGTTTATCTCTACGTAGTTAATAGAGATGTAAAGCTTAACCTGTGAGTCATCTAGTTATTTACCGTTCCAAGGAACCTGCGCTCCCTGATTTTTCAAAGGAAGACAAGGACATTGGCGACGTGTTTTTCGATGTGAGGGTTTTCAATCAACTCAACCTATCTGGCTTATATCACTCCTGAAAGTCGAACGTGCTTTCGACTGACGGTAGCGCTGTCGTAGCCGTCTGCAGGTTCTCCCCTTGCGGTAAGTATTTTGCCTATGCGATATCTCACTTGGTGGGTGCTATATTCCGGTATGTACTGCGTCGTCTTTCTGATTCTTTTACATAGGGAGGTGATTTTTCAACAATATATATTCGCCCTACGAGCGCTCCGTTGTCTCAGGCAACGACAATCGAGGACGATAAAGGCCGATTTCAAGATGAAGTGAAACGATTCAAGTTCTCGTCCGTCACATGGACGAAAGACTCCAAAGGCTTTCTTTACCAAGCACGTCATATCTCTCTTTCGTTCAATTTATCATGTGCCCTGACAAAGCCTAAAGCGGTTCCCTGCTCGAGACCCTAACGCTGAGAGAACTGCTGACCGAGATGCTATGATTTGTTACCATCGAATTGGAACGCCCCAATGTAAGGATGCGGTAACCCATAACCTTCATGAAACTAATCACATGCTCCAGCCGATGATATTATCGTGTACCAAGACAAAGAGCACCCAGAGTGGGTATTCGACACGTACGCCTCTGATGACGGAAAATATCTCTTCTTGTATCAGTATAAAAATACCGCAAAAGCAAGTCATTATTGTTTTATCGTAGGGGATTCAAGTAACCCTCTCGTTACCAGCTGAATTTTGTGTGGGTTACCGAGCTCGATGAAGGCGGGATCAAGCCTGAAATTCAGTGGCGAAAAATCATCAATGAATATGTGGCTGATTATAGCATGTCAAGATCTTGCCTTCGCTTCTCCATGATTTTTTTGTACTGACCCGAAATGAAGAATTACGAATCATGGGCCGCTGCTGTATGCCAGGACCAACCTGGATGCACCCCAATACAAGCTGGTTACTATCAACCTCTCGAAGGGTGGGGACCCAACCTTTGACCTGATTCCAGAGGCAAAGGATGCAAAGCTTGTCCAAGTTACCTGCGTCAACAGGGAATACTTTGTCGCTATCTATAAGCGCAACGTAATCCCAGCTATTTCCTTTATTTATTATCATGATACTAATGCGACGCATACAGGCCAAGGATGAAATCTATCTTTACTCCAAGGACGGCGCACAGCTAGAACGTCTGGCAGAAAATTTTGTTGGCGCTTCAACGATAGTAAACAGAGAAAAGCAGCACCATTTCTTCATTACGATGGCGGGATTTGACACGCCTGGTACCATTGCTCGCTACGACTTTACGGCTCCAGAATCACAGCGTTTTAGCATCCTTCGAACGACAAAGGTCAATGGGCTAAACCCAGATGAATTTGAGTCCAGGCAAGTCTGGTATGACAGTCATGACGGGATCAAGGTTCCTATGTTCATTGTTCGTCACAAATCGACGCAATTCGATGGAACGGCAGCGGCTATTCAATACGGTAACTTCTTGCTCTGCTGAAAGCATAGGAAATACACTCACAAGCATTGATAGGTTATGGTGGATTTGCTATTTCGGCGGATCCATTCTTCAGTCCAATTATCCTTACATTCCTGCAAATGTACGGTGCAATTCTCGCTGTTCCCAACATCAGGGGTGGGGGCGAATTCGGTGAGGAATGGCACAAGAGCGGAAGGCGAGAGAATAAGGTATATGAAAATATTTAACCCAAATCCAGTCAGTTAACCTTTCAAAGGGAAACAGTTTTGACGACTTCATTTCTGCTGCGTATGTCCTTGACCACCAAGTCCTATGATGACCCATGTTAACAAAGACCTCTTCCAAAGTCAATTTCTCGTCAAGAACAAGTACGCTGCCCCCGGTAAGGTGGCCATCAGCGGTGCATCTAATGGCGGTCAGACATTCATTACTGTCTTCTGTTTAACACACATCTCACGATGAAACCAGGTTTTTTGGTCTGCGGCTCCATAGTCCGAGCACCAGAAGGAACTTTCGGTGCGGCTATCGCCGAAGGCGGCGTTGCAGACCTCCTCAAGGTAAATATTTGAATTGTTGTCTATCGAATGCTAGCCCTCATGTTTCTGATTGATATATGCCTAGTTCTACAAATTTACCGGCGGTATGTTACGAGCCGTCTGTCCCCCCGCTAATCAGATTAAAATTGAATATCACTAGGAAAAGCTTGGACGAGCGAATACGGTGACCCCCGCGTTCCCGAGGACTTTGACTTTATCCATCCATTGTCTCCTTTACACAACGTACCTACCGACAAGGCTTTACCGGCCACATTACTCATGACCAATGCTGGTGAGCAGCTCCCACATGTTTGTATCACACCACCTGATCAAAGATCGGTCGTCAGCCGATGACCGTGTCGTTCCGATGCATTCGCTCAAGTTCATAGCGACACTTCAGCATAATTTGCCGCACAATCCAAACCCACTGCTGTTCCGCGTAGACAAGACTTGGTTGGGTCATGGCTATGGCAAGTCAACGGATAGGCAGTAAGTCGAATGTGCTGCCGGTCCATTCCGCTATTGATCGCTCTCTAGTATCAAGGACGCCGCTGATAAGTGGGGGTTTGTTGTGCAATCTCTAGGACTCGTGCGCAAGCAAGCGGCAGTGA

2: amino acid sequence

MPPTLWTTAKYPSARRSDHVDVYKSALKGEVHIPDPYQWLEGNSEEVDDWMTAQAAFTRAYLDQNQGSHELEDKFRANLNYAKFSAPTLLDDGHWYWFYNSGLQSQSVIYRSKEPALPDFSKEDKDIGDVFFDSNVLSTDGSAVVAVCRFSPCGKYFAYAISHLGGDFSTIYIRPTSAPLSQATTIEDDKGRFQDEVKRFKFSSVTWTKDSKGFLYQRFPARDPNAERTADRDAMICYHRIGTPQSDDIIVYQDKEHPEWVFDTYASDDGKYLFLYQYKNTAKLNFVWVTELDEGGIKPEIQWRKIINEYVADYSIITNHGPLLYARTNLDAPQYKLVTINLSKGGDPTFDLIPEAKDAKLVQVTCVNREYFVAIYKRNAKDEIYLYSKDGAQLERLAENFVGASTIVNREKQHHFFITMAGFDTPGTIARYDFTAPESQRFSILRTTKVNGLNPDEFESRQVWYDSHDGIKVPMFIVRHKSTQFDGTAAAIQYGYGGFAISADPFFSPIILTFLQMYGAILAVPNIRGGGEFGEEWHKSGRRENKGNSFDDFISAAQFLVKNKYAAPGKVAISGASNGGFLVCGSIVRAPEGTFGAAIAEGGVADLLKFYKFTGGKAWTSEYGDPRVPEDFDFIHPLSPLHNVPTDKALPATLLMTNAADDRVVPMHSLKFIATLQHNLPHNPNPLLFRVDKTWLGHGYGKSTDRHIKDAADKWGFVVQSLGLVRKQAAV
