# Supplementary material for: Genome of lethal Lepiota venenata and insights into the evolution of toxin-biosynthetic genes
Source: BMC Genomics. 2019 Mar 8;20:198. doi: 10.1186/s12864-019-5575-7 (PMC6408872; doi:10.1186/s12864-019-5575-7)
Supplement: Supplementary file 3 — Accession numbers of prolyl oligopeptidase gene and amino acid sequences included in the phylogenetic study. (DOCX 25 kb) [file 12864_2019_5575_MOESM3_ESM.docx]

Accession numbers of prolyl oligopeptidase gene and amino acid sequences included in the phylogenetic study.

| Taxon | Strain | Prolyl Oligopeptidase | Source | Amino Acids | Coding Sequence |
| --- | --- | --- | --- | --- | --- |
| *Agaricus bisporus* var*. bisporus* | H97 | POP | JGI | 219134 |  |
| *Agrocybe pediades* | AH 40210 | POP | JGI | 736488 |  |
| *Anomoporia bombycina* | ATCC 64506 | POP | JGI | 1333562 |  |
| *Antrodia sinuosa* | LB1 | POP | JGI | 705706 |  |
| *Artolenzites elegans* | CIRM-BRFM 1663 | POP | JGI | 887929 |  |
| *Auricularia subglabra* | – | POP | JGI | 1169635 |  |
| *Auriculariopsis ampla* | NL-1724 | POP | JGI | 534627 |  |
| *Amanita bisporigera* | – | POPA | NCBI | ADN19204 | HQ225840 |
| *Amanita bisporigera* | – | POPB | NCBI | ADN19205 | HQ225841 |
| *Amanita muscaria* | Koide | POPA | JGI | 74086 |  |
| *Amanita pallidorosea* | – | POPA | Genome | – |  |
| *Amanita pallidorosea* | – | POPB | Genome | – |  |
| *Amanita phalloides* | – | POPA | Genome | – |  |
| *Amanita phalloides* | – | POPB | Genome | – |  |
| *Amanita rimosa* | – | POPA | Genome |  |  |
| *Amanita rimosa* | – | POPB | Genome |  |  |
| *Amanita subjunquillea* | – | POPA | Genome | – |  |
| *Amanita subjunquillea* | – | POPB | Genome | – |  |
| *Amanita thiersii* | Skay4041 | POPA | JGI | 193040 |  |
| *Beauveria bassiana* | ARSEF 2860 | POP | JGI | 1657 |  |
| *Bolbitius vitellinus* | SZMC-NL-1974 | POP | JGI | 1302751 |  |
| *Calocera cornea* | – | POP | JGI | 490308 |  |
| *Ceraceosorus bombacis* | MCA 4658 | POP | JGI | 333060 |  |
| *Cerrena unicolor* | – | POP | JGI | 313626 |  |
| *Clitocybe gibba* | IJFM A 808 | POP | JGI | 1443961 |  |
| *Colletotrichum nymphaeae* | SA-01 | POP | JGI | 1020605 |  |
| *Conocybe apala* | – | POP | NCBI | ACQ65797 | FJ906819 |
| *Coprinopsis cinerea* | okayama7#130 | POP | NCBI | XP_001841244 | XM_001841192 |
| *Cortinarius glaucopus* | AT 2004 276 | POP | JGI | 7259917 |  |
| *Crepidotus variabilis* | CBS 506.95 | POP | JGI | 870504 |  |
| *Crucibulum laeve* | CBS 166.37 | POP | JGI | 718333 |  |
| *Cyathus striatus* | AH40144 | POP | JGI | 1424557 |  |
| *Cytidiella melzeri* | FP 102339 | POP | JGI | 1412396 |  |
| *Dichomitus squalens* | LYAD-421 SS1 | POP | JGI | 160828 |  |
| *Fibulorhizoctonia* sp. | CBS 109695 | POP | JGI | 740187 |  |
| *Fomitiporia mediterranea* | – | POP | JGI | 141658 |  |
| *Galerina marginata* | – | POPA | JGI | 70906 |  |
| *Galerina marginata* | – | POPB | JGI | 146341 |  |
| *Gloeophyllum trabeum* | ATCC 11539 | POP | NCBI | XP_007862595 | XM_007864404 |
| *Gymnopilus chrysopellus* | PR-1187 v1.0 | POP | JGI | 1688417 |  |
| *Hebeloma cylindrosporum* | h7 | POP | JGI | 444548 |  |
| *Heliocybe sulcata* | OMC 1185 | POP | JGI | 1641899 |  |
| *Hydnomerulius pinastri* | – | POP | JGI | 174977 |  |
| *Hydnopolyporus fimbriatus* | CBS384.51 | POP | JGI | 983183 |  |
| *Hypsizygus marmoreus* | 51987-8 | POP | NCBI | KYQ30898 | LUEZ01000114 |
| *Laccaria bicolor* | – | POP | JGI | 303722 |  |
| *Lentinus tigrinus* | – | POP | JGI | 581405 |  |
| *Leiotrametes* sp. | BRFM 1775 | POP | JGI | 1337173 |  |
| *Lepiota subincarnata* | – | POPB | JDW* | – |  |
| *Lepiota subincarnata* | – | POPB | JDW* | – |  |
| *Lepiota venenata* | – | POPB | Genome | – |  |
| *Lepiota venenata* | – | POPC | Genome | – |  |
| *Lepista nuda* | CBS 247.69 | POP | JGI | 1271729 |  |
| *Leucogyrophana mollusca* | KUC20120723A-06 | POP | JGI | 1128529 |  |
| *Macrolepiota fuliginosa* | MF-IS2 | POP | JGI | 799170 |  |
| *Malassezia pachydermatis* | CBS 1879 | POP | NCBI | KOS13970 | LGAV01000004 |
| *Marssonina brunnea* f. sp. '*multigermtubi'* | MB_m1 | POP | JGI | 1261 |  |
| *Metarhizium robertsii* | ARSEF 23 | POP | JGI | 9962 |  |
| *Ophiocordyceps sinensis* | CO18 | POP | NCBI | EQL04271 | KE652172 |
| *Panus rudis* | PR-1116ss-1 | POP | JGI | 1587789 |  |
| *Paxillus adelphus* | Ve08.2h10 | POP | JGI | 30278 |  |
| *Phanerochaete carnosa* | HHB-10118-Sp | POP | JGI | 191914 |  |
| *Pisolithus tinctorius* | Marx 270 | POP | JGI | 969495 |  |
| *Pleurotus ostreatus* | PC 15 | POP | JGI | 1092697 |  |
| *Plicaturopsis crispa* | – | POP | JGI | 170617 |  |
| *Pluteus cervinus* | NL-1719 | POP | JGI | 759937 |  |
| *Polyporus brumalis* | BRFM 1820 | POP | JGI | 1399521 |  |
| *Rhizoctonia solani* | AG-1 IB | POP | JGI | 7338 |  |
| *Rhizopogon vinicolor* | AM-OR11-026 | POP | JGI | 794021 |  |
| *Schizophyllum commune* | Tattone D | POP | JGI | 421410 |  |
| *Serpula himantioides* | SHA21-2 | POP | JGI | 40692 |  |
| *Suillus decipiens* | EM49 | POP | JGI | 1139982 |  |
| *Trametes cingulata* | BRFM 1805 | POP | JGI | 1559098 |  |
| *Trametes versicolor* | – | POP | JGI | 117177 |  |
| *Xerocomus badius* | 84.06 | POP | JGI | 1422158 |  |
| *Wolfiporia cocos* | MD-104 SS10 | POP | JGI | 89370 |  |
| *Stereum hirsutum* | FP-91666 SS1 | POP | JGI | 59870 |  |

* Provided by Jonathan D. Walton at Michigan State University
